# Boosting of photocatalytic hydrogen evolution via chlorine doping of polymeric carbon nitride

**DOI:** 10.3762/bjnano.12.38

**Published:** 2021-05-19

**Authors:** Malgorzata Aleksandrzak, Michalina Kijaczko, Wojciech Kukulka, Daria Baranowska, Martyna Baca, Beata Zielinska, Ewa Mijowska

**Affiliations:** 1 Nanomaterials Physicochemistry Department, Faculty of Chemical Technology and Engineering, West Pomeranian University of Technology, Szczecin, Piastow Ave. 42, 71-065 Szczecin, Poland

**Keywords:** chlorine, doping, hydrogen evolution reaction, photocatalysis, polymeric carbon nitride

## Abstract

Chlorine is found to be a suitable element for the modification of polymeric carbon nitride properties towards an efficient visible-light photocatalytic activity. In this study, chlorine-doped polymeric carbon nitride (Cl-PCN) has been examined as a photocatalyst in the hydrogen evolution reaction. The following aspects were found to enhance the photocatalytic efficiency of Cl-PCN: (i) unique location of Cl atoms at the interlayers of PCN instead of on its π-conjugated planes, (ii) slight bandgap narrowing, (iii) lower recombination rate of the electron–hole pairs, (iv) improved photogenerated charge transport and separation, and (v) higher reducing ability of the photogenerated electrons. The above factors affected the 4.4-fold enhancement of the photocatalytic efficiency in hydrogen evolution in comparison to the pristine catalyst.

## Introduction

Currently, the biggest problems of civilization seem to be the global energy crisis and environmental pollution. Both of these problems are directly related to each other. The pollution of our planet is mainly due to fossil fuels used in the energy industry, the combustion of which generates CO_2_ emissions.

The ideal solution of these problems appears to be the use of photocatalysis. The solar light, as a driving force, has been widely used in different fields, such as water in water-splitting to generate hydrogen [1–4], environmental remediation [5–6], decomposition of organic pollutants [7], CO_2_ reduction into hydrocarbon fuels [8–10], disinfection [11–12], and selective organic transformations [13–14].

One of the most studied catalysts is polymeric carbon nitride (PCN). This graphite-like semiconductor polymer, as a metal-free and visible-light-responsive photocatalyst, has attracted dramatically growing attention in the field of visible-light-induced hydrogen evolution reaction (HER). It is characterized by facile synthesis, easy functionalization, attractive electronic band structure, and photocatalytic activity [15–17]. Furthermore, it exhibits high thermal and chemical stability during photocatalytic reactions in the aqueous phase [18]. Unfortunately, its catalytic performance is mainly constrained by several typical challenges, which are the low density of reactive sites, nonresponse in the long-wavelength region, sluggish kinetics, and high recombination of photoexcited electron–hole pairs [19–21].

Tremendous efforts have been made in order to increase the photocatalytic activity of PCN materials by optimizing their nanostructure and improving their chemical surface texture. Three of the most popular modifications are: (i) coupling with other semiconductors [22–23], (ii) self-optimization of the crystal structure [24–25], and (iii) doping with heteroatoms [26–27]. Therefore, PCN is called the "holy grail" because it is believed that its modifications will result in obtaining a highly efficient HER under visible light conditions [28–29].

One of the most effective methods to modify the electronic structure and improve photocatalytic properties, among so many options, seems to be non-metallic doping [30–33]. For instance, Ma et al. found that the doping of PCN with the P atom may promote the mobility of the charge carrier and facilitate the separation of the photogenerated electron–holes [34]. Another research group found that their prepared fluorinated carbon nitride has a photocatalytic activity 20.8 times higher than that of pristine PCN [30]. Wang et al. studied the photoactivity of PCN doped with S in the CO_2_ reduction reaction. The yield of CH_3_OH over the unit area of the photocatalyst was almost 2.5 times higher than of pristine PCN [35].

Recently, co-doping of g-C_3_N_4_ with two non-metallic elements has been also studied. This strategy can enhance photocatalysis by imparting additional merits of each of the co-dopants of the photocatalyst. Polymeric carbon nitride has been co-doped with B/F [36], S/P [37], or C/P [38]. Yi et al. showed that PCN co-doped with S and Cl had better catalytic efficiency in the degradation of rhodamine B and 4-nitrophenol under visible light compared to catalysts doped with one heteroatom [39]. Other studies showed that S- and P-doped photocatalysts showed significantly increased photocatalytic activity in the degradation of methylene blue under visible light compared to bulk PCN. The improvement was attributed to lone-pair electron delocalization, efficient charge separation, favorable retention of the crystal structure, and light-harvesting extension [37].

Here, a new procedure of PCN doping with chlorine will be revealed. The photocatalytic activity of the prepared materials was investigated in a water-splitting reaction with hydrogen evolution under simulated solar light. A series of microscopic and spectroscopic techniques have been used to characterize the morphology, chemical structure, optical, photophysical, and electrical properties of the obtained carbon nitrides.

## Results and Discussion

**Figure 1:**
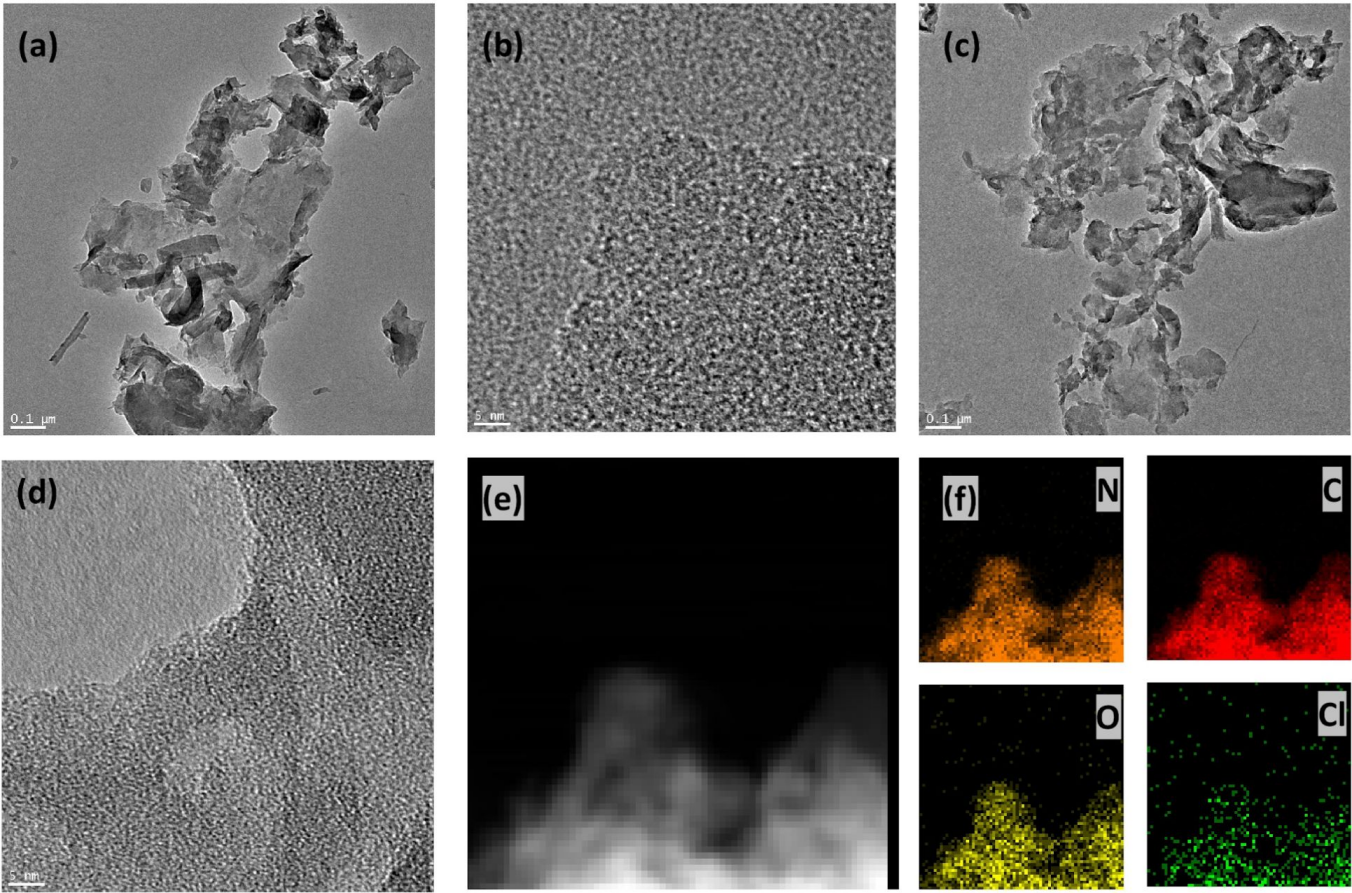
TEM images of PCN (a, b) and Cl-PCN (c, d). Scanning transmission electron microscopy image of Cl-PCN (e) and energy-dispersive X-ray spectroscopy (EDX) elemental mappings of N, C, O, and Cl in Cl-PCN (f).

The detailed analysis of the morphology of the prepared materials, presented in Figure 1, was performed by transmission electron microscopy (TEM). The images of pristine PCN demonstrate the layered structure with a tendency to fold and aggregate. They also show several stacking layers, indicating the planar graphitic-like structure. After Cl-doping, a relatively uniform-stacked petal-like nanosheet structure with small pores on the surface was formed (Figure 1). A higher magnification shows that in-plane mesopores of tens of nanometers are randomly distributed on the carbon nitride nanosheets (Figure 1d). The Cl-PCN porous structure allows for the catalyst to have a higher specific surface area and more active sites, which can simultaneously promote mass transfer and charge separation in nanodomains, thus optimizing the π-conjugated system for photochemical applications [40–41]. Furthermore, elemental mapping of nitrogen, carbon, oxygen, and chlorine in Cl-PCN was performed and showed homogeneous distribution of all elements in the sample.

**Figure 2:**
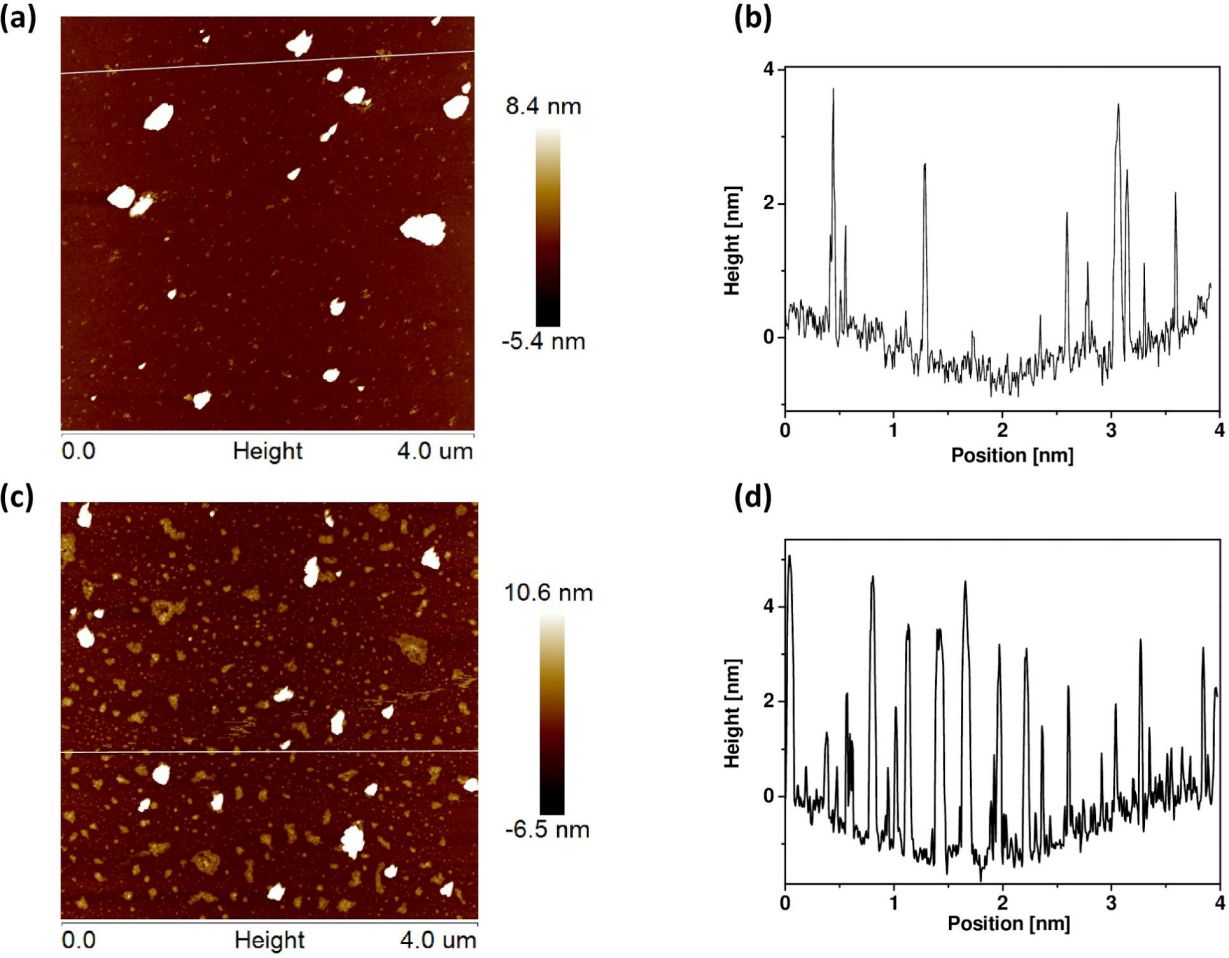
AFM images and height profile of PCN (a, b) and Cl-PCN (c, d).

As analyzed via atomic force microscopy (AFM, Figure 2a and Figure 2b) the as-prepared PCN aggregated as large sheets with thickness ranging from 1 to 4 nm (corresponding to 3–11 atomic layers). In comparison, the Cl-doped PCN (Figure 2c and Figure 2d) revealed a thickness range from 0.5 to 5 nm (corresponding to 2–14 atomic layers) with the dominating fraction ranging from 2 to 4 nm. The slight enlargement of the lattice parameters can be explained by the unique location of Cl atoms at the interlayers of PCN and not on its π-conjugated planes as it is in the case with other commonly used metal/non-metal (Cu, Ni, C, N or O) modifications [42–46].

**Figure 3:**
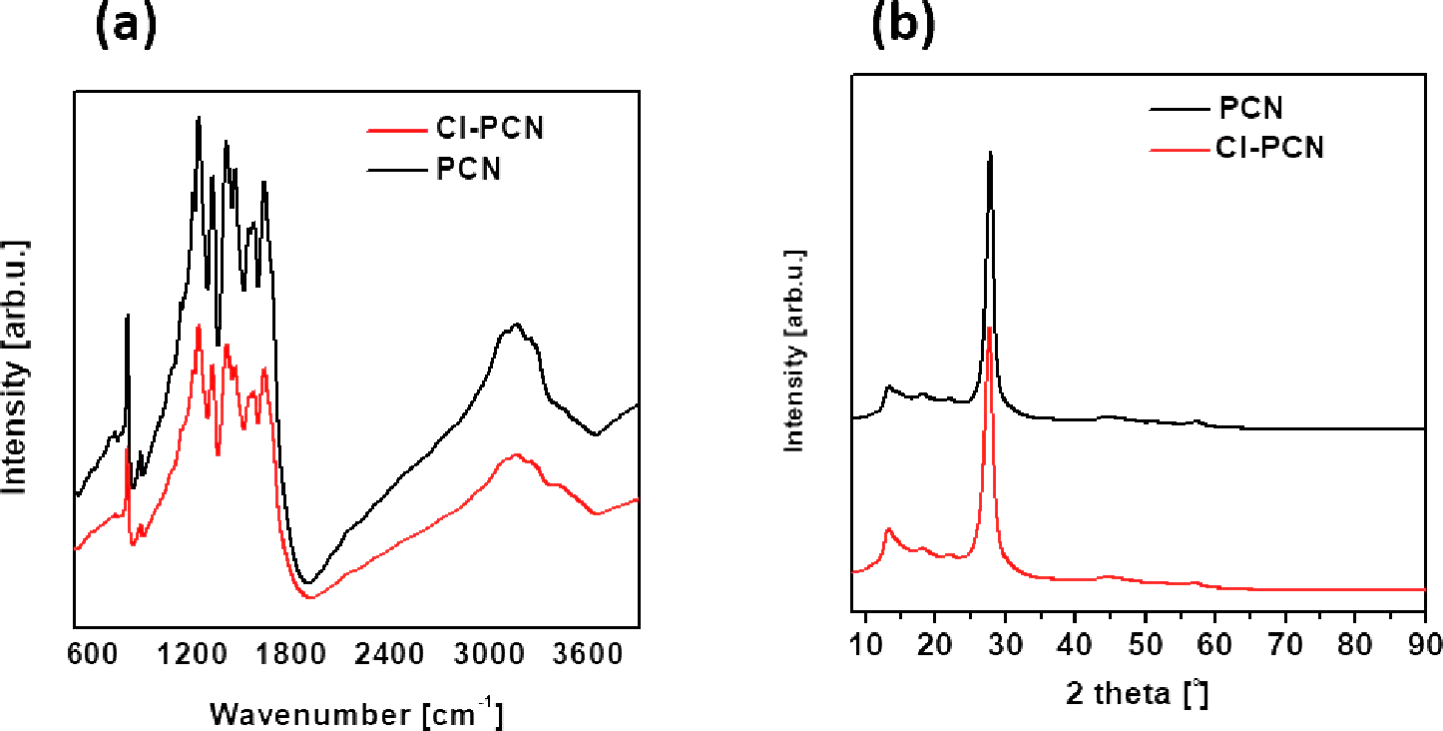
(a) FTIR spectra and (b) XRD patterns of PCN and Cl-PCN.

Fourier-transform infrared (FTIR) spectroscopy was used to obtain the molecular structure information of the carbon nitride materials. The FTIR absorption analysis was recorded in the spectral range of 600–3600 cm^−1^ to examine the surface of the prepared materials (Figure 3a). The FTIR spectra of both samples (before and after doping) reveal that the positions of the vibration peaks are nearly the same, indicating a similar molecular structure of the samples which is well maintained even after chemical doping of Cl. The signal at 810 cm^−1^ represents the *s*-triazine ring models, which correspond to the condensed CN heterocycles. The intense signal between 1200 and 1600 cm^−1^ is indicative of the characteristic stretching vibration of the CN heterocycles [47–49]. To be more specific, the peaks at 1241, 1318, and 1425 cm^–1^ are assigned to the aromatic C–N stretching [50–51] while the peaks at 1572 and 1637 cm^−1^ correspond to C=N stretching [52]. The broad peaks in the range of 3000–3600 cm^−1^ correspond to uncondensed terminal amino groups (–NH_2_ or =NH) [53–54]. The spectra do not show Cl-containing functional groups, which can be attributed to their relatively low amount and the signal may be overlapped by the CN vibration.

The X-ray diffraction (XRD) patterns (Figure 3b) showed that both samples displayed a similar crystalline phase with two characteristic peaks at approx. 27.38° and 13.28°, corresponding to (002) and (100) crystal planes for PCN, respectively. The (002) peak is associated with the typical interplanar stacking peak of conjugated aromatic structures, whereas the (100) peak is attributed to the in-plane packing motif of the tri-*s*-triazine units. The shift from 27.38° to 27.30° is caused by the increased internal distance of PCN by Cl doping, which is in good agreement with AFM data and suggests that Cl is located at the interlayers of carbon nitride. Moreover, the XRD and FTIR analyses confirmed that the Cl modification resulted in the maintenance of the chemical skeleton [43–47].

**Figure 4:**
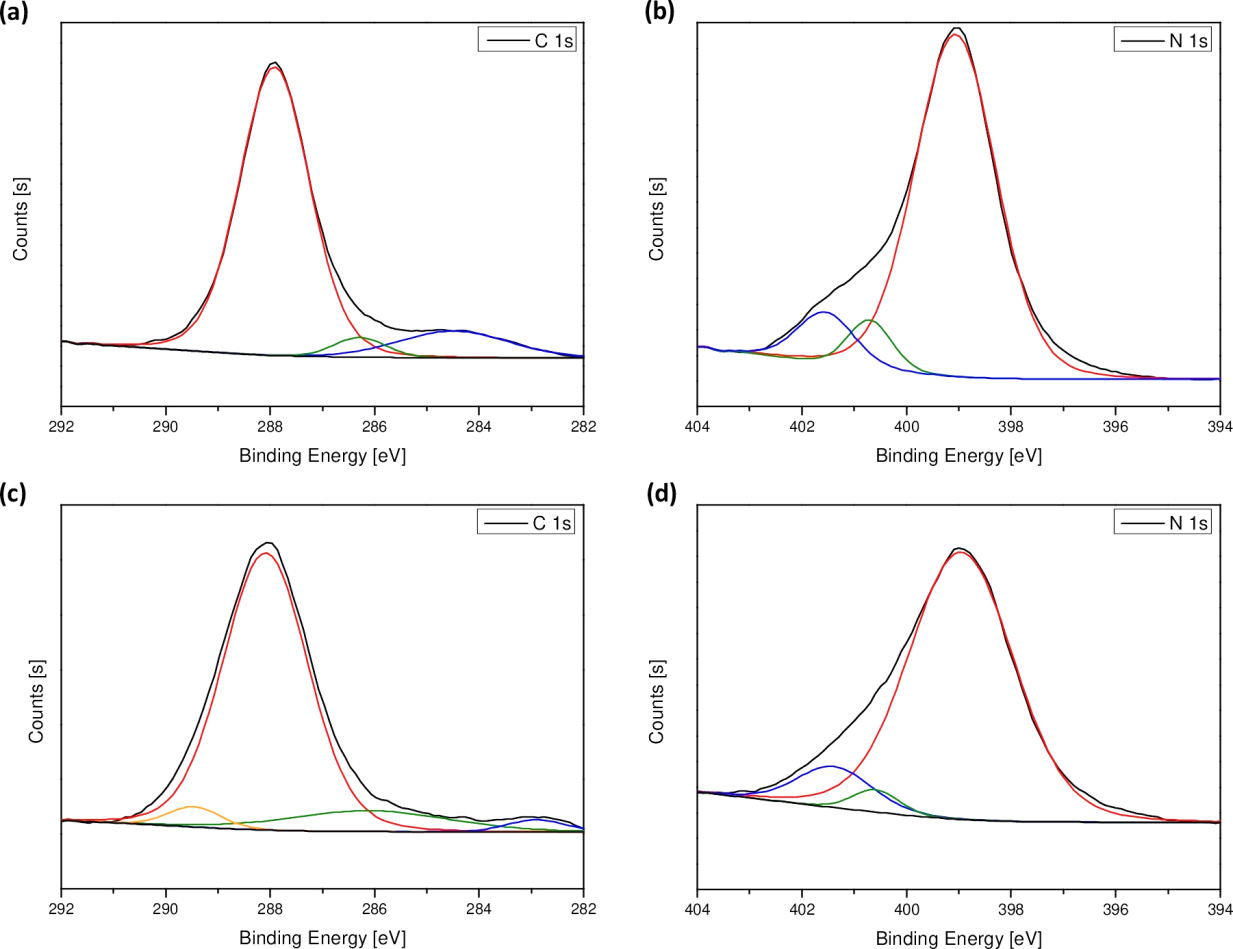
C 1s and N 1s XPS spectra of polymeric carbon nitride (a, b) and Cl-PCN (c, d).

The chemical composition and relative atomic percentages of the obtained materials were analyzed by X-ray photoelectron spectroscopy (XPS). The XPS spectra revealed that the samples are composed of carbon, nitrogen, and oxygen. Additionally, the chlorine signal was detected in the doped sample. The atomic concentration of the elements was calculated assuming a homogeneous distribution in the analyzed surface layer and it is given in Table 1. The obtained results show that melamine polycondensation with 2-chloro-4,6-diamino-1,3,5-triazine (CDATA) increased the amount of carbon whereas it decreased the amount of nitrogen. Simultaneously, a slight increase in the atomic concentration of oxygen was observed. The XPS spectra revealed 0.18 atom % of chlorine in Cl-PCN. The detailed analysis of the chemical components carbon and nitrogen was done by applying the peak-fitting procedure to the N 1s and C 1s spectra of the obtained samples and the results are shown in Figure 4 and in Table 2. The type of binding energy as well as the relative contribution of each component to the total area under the peak were calculated. Peaks located at approx. 399 and 288 eV are assigned to signals from N 1s and C 1s, respectively. The C 1s region consists of three contributions which are associated to C–C, N–C=N, and C–NH*_x_*. The N 1s region consists of three contributions which are associated to C–N_3_ (N_3_C), N–C=N (N_2_C), and N–H*_x_*. The sample Cl-PCN presents an additional contribution at 289 eV, which is related to C–Cl. It can be observed that as a result of chlorine doping the content of N–H*_x_*/C–NH*_x_* increases compared to the starting material. In the case of Cl-PCN, the content of N–C=N/N_2_C bonds also increases. On the other hand, the amount of C–C/C=C/N_3_C significantly decreases.

**Table 1:** C, N, O, and Cl atomic concentration in PCN and Cl-PCN.

Sample	C (atom %)	N (atom %)	O (atom %)	Cl (atom %)

PCN	36	63.58	0.42	–
Cl-PCN	36.86	62.31	0.65	0.18

**Table 2:** Chemical composition of PCN and Cl-PCN calculated from the peak-fitting procedure applied to the N 1s and C 1s spectra of the samples.

Sample	N–C=N (atom %)	C–NH*_x_* (atom %)	C–C/C=C (atom %)	C–Cl (atom %)	N_2_C (atom %)	N–H*_x_* (atom %)	N_3_C (atom %)

PCN	83.61	4.23	12.16	–	84.77	8.73	6.50
Cl-PCN	82.13	2.49	11.67	3.71	87.23	9.54	3.23

**Figure 5:**
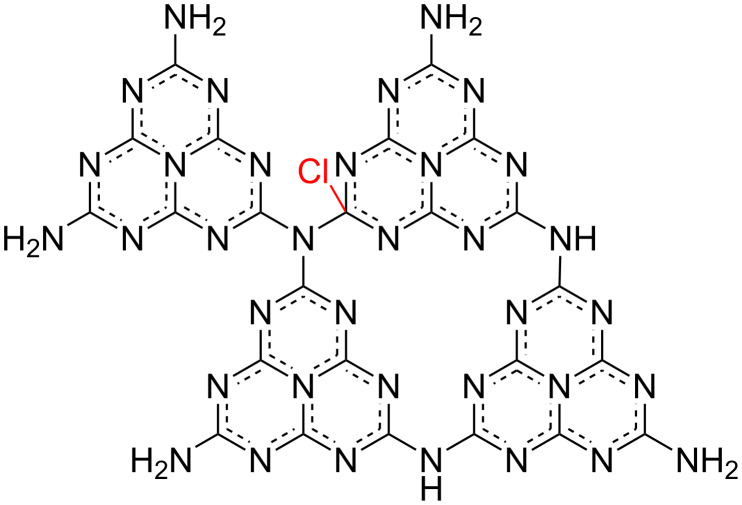
Structure of chlorine-doped polymeric carbon nitride.

The XPS analysis indicates a successful incorporation of chlorine into the polymeric carbon nitride network. Without the doping agent melamine, thermal polycondensation leads to the formation of melon. We suppose that the presented synthesis procedure results in the substitution of the melamine molecule with 2-chloro-4,6-diamino-1,3,5-triazine to form Cl-doped melon, followed by further polycondensation leading to chlorine-doped polymeric carbon nitride. The substitution has an effect on the appearance of C–Cl bonds in the PCN structure where chlorine atoms are located between the carbon nitride layers, as reported by other groups [39,55]. After doping with Cl, the contribution of C–NH*_x_* bonds to the XPS signal decreased from 4.23% to 2.49% and that of N_3_–C bonds from 6.50% to 3.23%, with a simultaneous increase of the N_2_–C contribution from 84.77% to 87.23%. The schematic representation of the as-synthesized material structure is shown in Figure 5.

**Figure 6:**
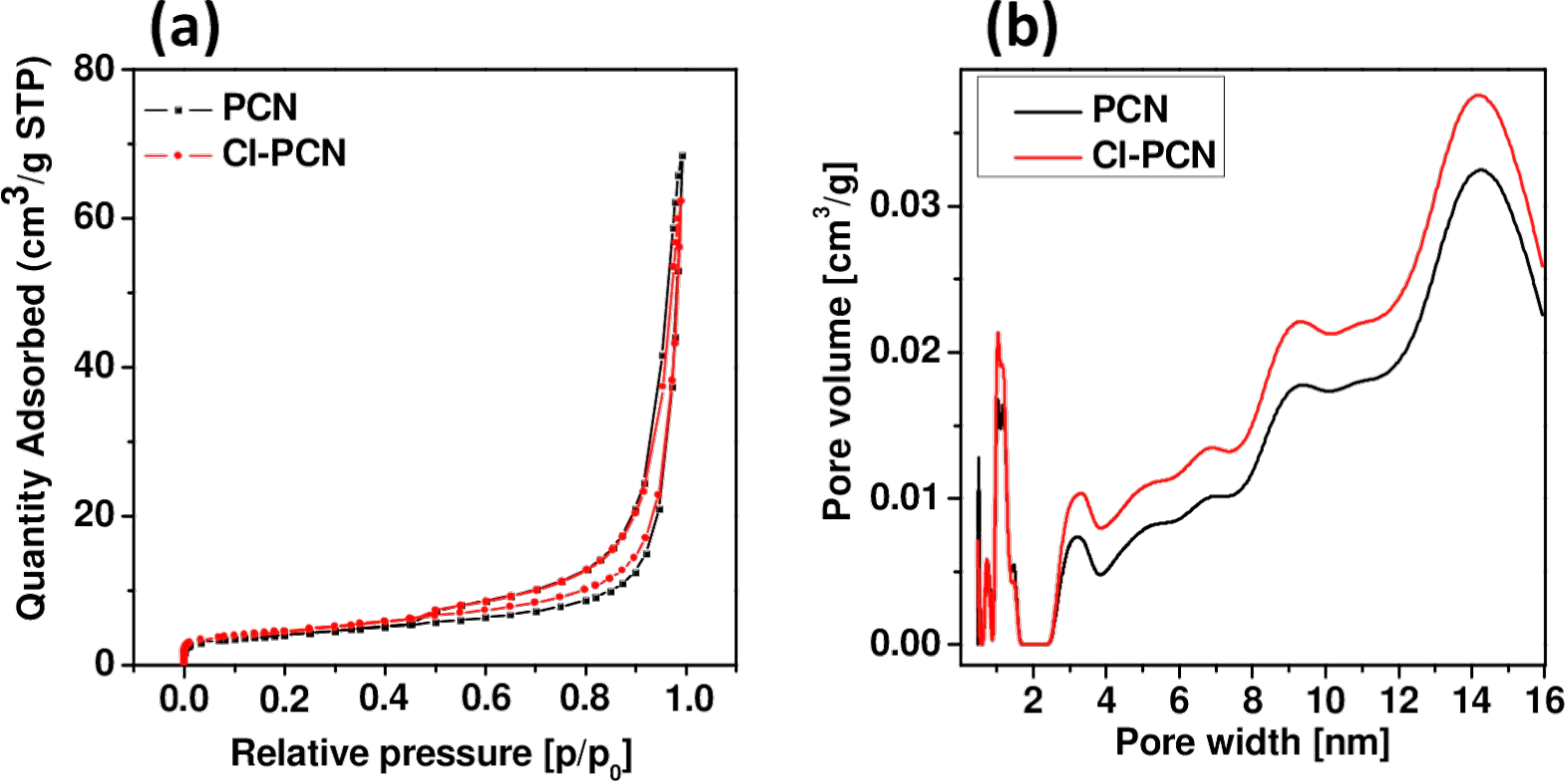
(a) Adsorption–desorption isotherms and (b) density functional theory (DFT) applied to the adsorption isotherms to obtain pore–size distributions of PCN and Cl-PCN.

The porosity of polymeric carbon nitride and PCN doped with chlorine was tested by the N_2_ adsorption–desorption experiment. The typical IV isotherms with H3 hysteresis loops are observed in the samples, which is typical of mesoporous materials (Figure 6). The hysteresis loops, pore–size distribution curves, and average pore diameter for both samples are similar. The proportion of micropores is small and the samples contain mainly mesopores. The sample modified by chlorine presents a slightly higher Brunauer–Emmet–Teller (BET) surface area, average pore diameter, and lower total pore volume. The details of the BET surface area, T-plot analysis results for micropore area, external surface area, total pore volume, and average pore diameter of PCN and Cl-PCN are presented in Table 3.

**Table 3:** BET surface area, T-plot analysis results for micropore area, external surface area, total pore volume, and average pore diameter of PCN and Cl-PCN.

Sample	PCN	Cl-PCN

BET surface area [m^2^/g]	14.43 ± 0.02	16.27 ± 0.01
T-plot micropore area [m^2^/g]	2.06	1.94
T-plot external surface area [m^2^/g]	12.37	14.32
total pore volume [cm^3^/g]	0.106	0.096
average pore diameter [nm]	5.65	5.76

**Figure 7:**
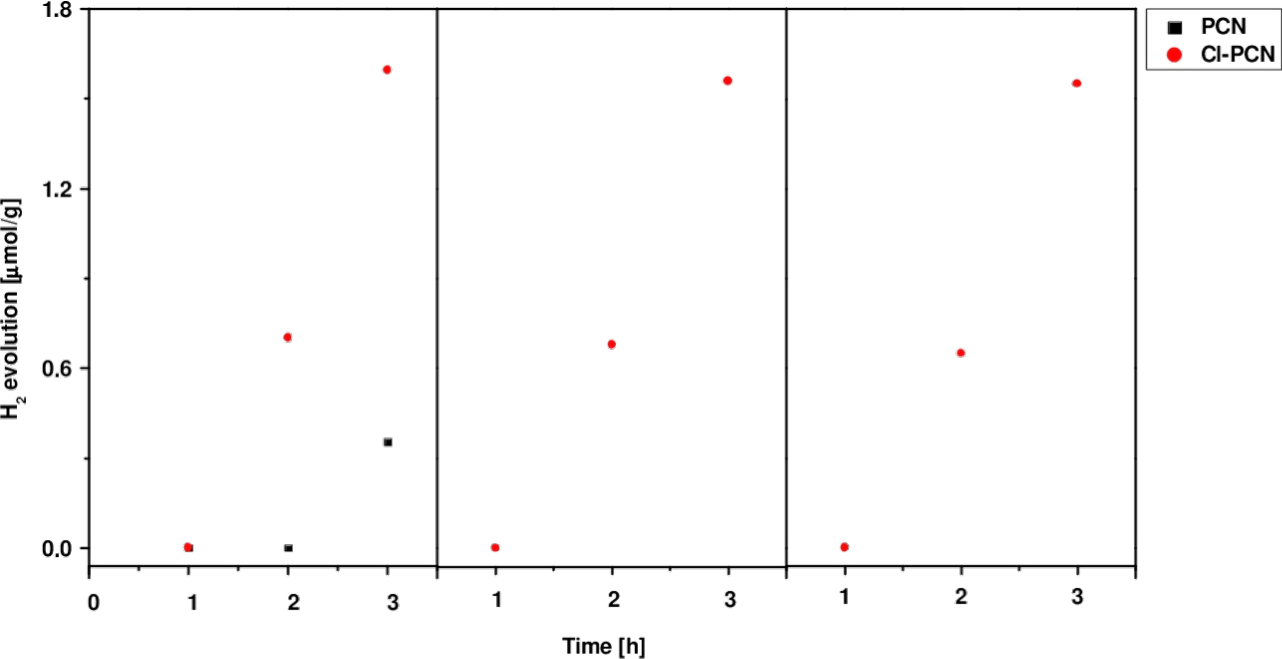
H_2_ evolution rate catalyzed by PCN and Cl-PCN.

The results of the photocatalytic hydrogen generation process under simulated solar light irradiation is presented in Figure 7. It is clear that the designed modification of the samples strongly boosts the photocatalytic efficiency. The hydrogen evolution of Cl-PCN was approx. 4.4 times higher after 3 h in relation to unmodified PCN. Therefore, chlorine doping is a reasonable strategy towards better photocatalytic hydrogen generation ability. To examine the stability of the photocatalytic activity of the Cl-doped carbon nitride, a recycle test has been performed. It revealed a decrease of approximately 2% in the H_2_ evolution rate after three cycles, indicating the stability of the catalyst.

Table 4 presents a comparative study of Cl-PCN with catalysts doped with Cl and other elements which have been reported in the literature. The table presents a broad range of the enhancement factor of the hydrogen evolution rate after PCN doping. Among the presented doping procedures, S- and Cl-doping were found to enhance HER of PCN more significantly [45,56].

**Table 4:** Comparative study of the photocatalytic hydrogen evolution of Cl- PCN and other carbon nitride catalysts doped with other elements.

Doping element	PCN precursor	Light	Enhancement factor of HER over the reference sample	Ref.

P	melamine	>420 nm	4	[57]
P	melamine	>420 nm	1.7	[58]
P	melamine	>420 nm	9.5	[59]
P	melamine	>420 nm	8.6	[60]
S	melamine	>420 nm	1.9	[58]
S	dicyandiamide	>420 nm	8	[61]
S	urea	>420 nm	30	[56]
B	melamine	>420 nm	2.7	[58]
B	dicyandiamide	>420 nm	12	[62]
B	melamine, urea	>420 nm	2.4	[63]
O	melamine	>420 nm	2	[64]
F	melamine	>420 nm	2.7	[65]
C	melamine	>420 nm	1.4	[66]
I	melamine	>420 nm	9	[67]
I	DCDA	>420 nm	2	[68]
I	DCDA	>420 nm	2	[69]
Br	urea	>420 nm	2	[27]
Br	urea	>420 nm	3.6	[70]
Cl	melamine	>420 nm	19.2	[45]
Cl	melamine	solar	4.4	this study
Mg/Cl	melamine	>420 nm	8.8	[55]

**Figure 8:**
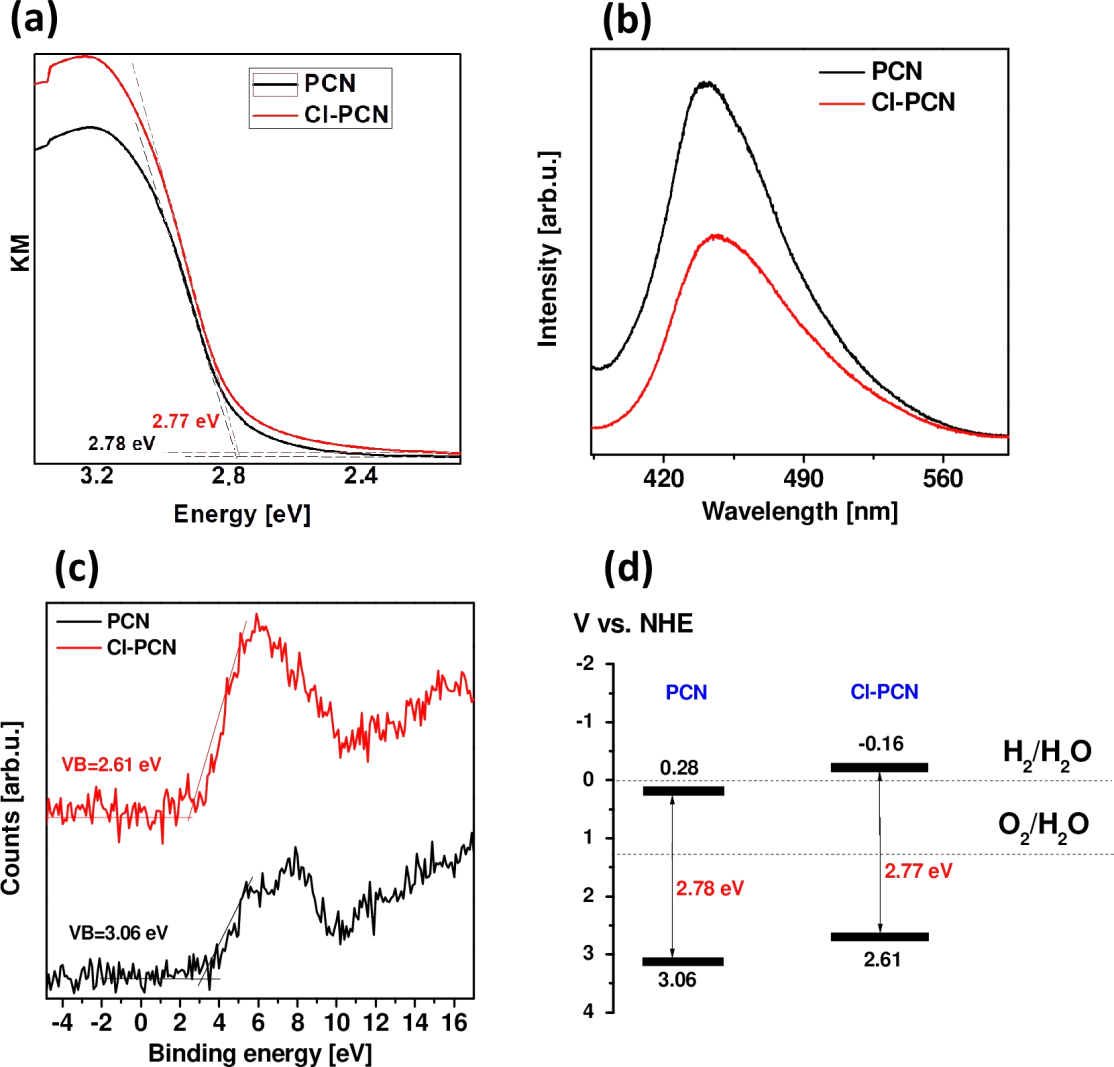
(a) DRS spectra, (b) PL emission spectra, (c) valence band (VB) XPS spectra, and (d) band diagram of PCN and Cl-PCN.

To explain the phenomenon of the enhanced photocatalytic H_2_ evolution after Cl-doping more studies have been conducted. The optical properties of PCN and Cl-PCN were investigated via UV–vis diffuse reflectance spectroscopy (DRS) and photoluminescence (PL) emission spectroscopy. Figure 8a shows the Kubelka–Munk function curves of the fabricated materials. The bandgap is 2.78 and 2.77 eV for PCN and Cl-PCN, respectively, indicating that the Cl-doping had no significant effect on the bandgap shift. This might be attributed to the low content of chlorine atoms in the material.

The PL spectra of PCN and Cl-PCN are presented in Figure 8b. The emission peak of PCN is located at approx. 440 nm, which is in accordance with the optical bandgap defined by the DRS measurement. Chlorine doping caused a slight redshift of the emission peak. Moreover, a reduction in the peak intensity was found, indicating a lower recombination rate of the electron–hole pairs, which is attractive in the photocatalytic process [44]. It shows that chlorine doping improves visible-light harvesting with PCN and promotes visible-light photocatalytic activity [55].

To estimate the valence band position of PCN and Cl-PCN, VB XPS spectra were measured and are presented in Figure 8c. Furthermore, the conduction band (CB) position of the samples was calculated from the formula *E*_CB_ = *E*_g_ − *E*_VB_, and the band diagram is presented in Figure 8d. The VB and CB positions to the more positive potential after chlorine doping is shifted, indicating strengthened reducibility of electrons in CB. This is one of the factors influencing the enhanced photocatalytic activity in the hydrogen evolution reaction.

**Figure 9:**
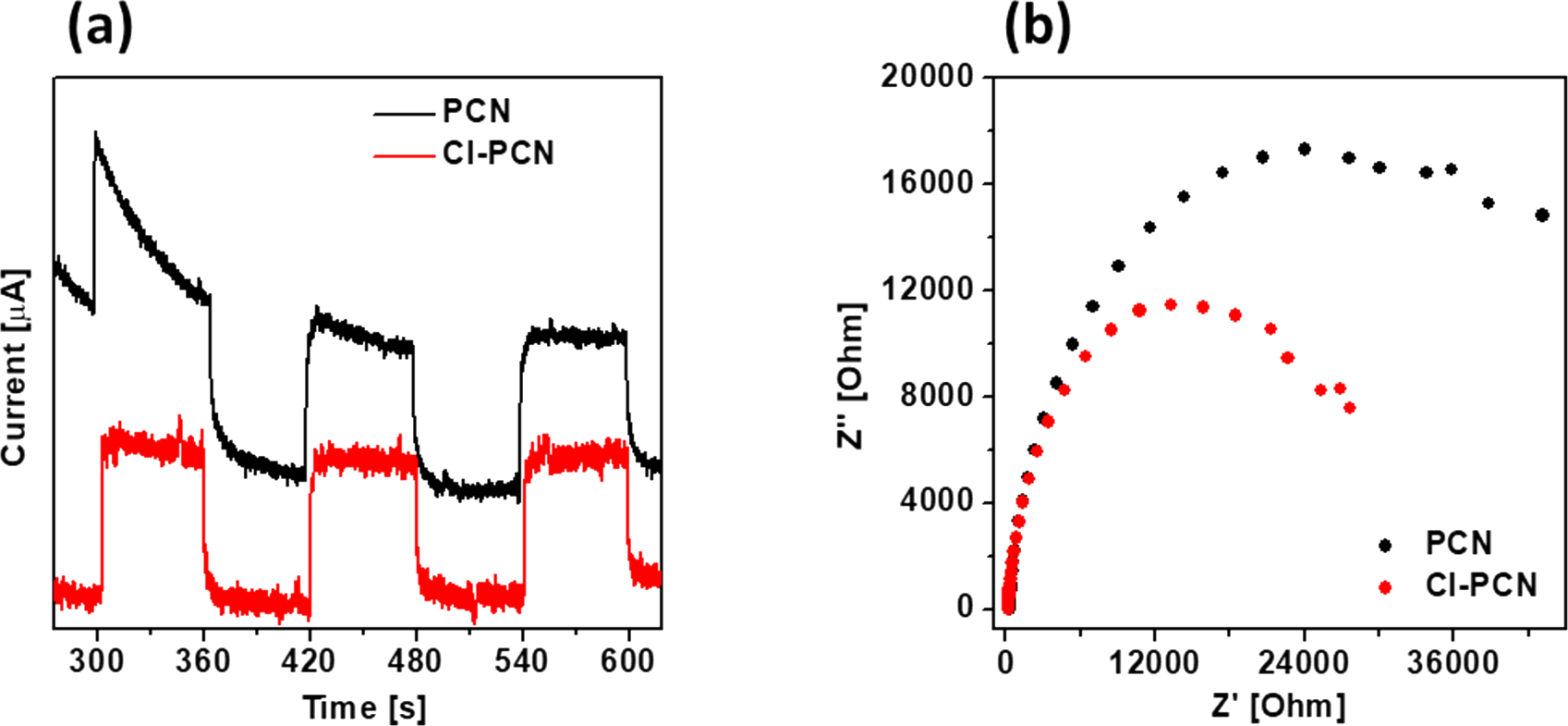
(a) Photocurrent response and (b) EIS spectra of PCN and Cl-PCN.

Figure 9a shows the transient photocurrent response of PCN and Cl-PCN. One can observe two-fold enhancement of the photocurrent response after chlorine doping of PCN. It demonstrates an improved generation of electron–hole pairs and better transportation of the charge carriers after modification. After three cycles of light on–off, the performance of both electrodes tends to stabilize, indicating that the photocatalysts are stable under visible-light irradiation [55].

The measurements obtained from electrochemical impedance spectroscopy (EIS) are shown in Figure 9b. It is known that the arc radius of the EIS spectrum is related to the charge-transfer resistance at the electrode–electrolyte interface [71]. The EIS arc radius of Cl-PCN is smaller than that of PCN. Its impedance is reduced compared to PCN, indicating that Cl doping decreased the charge-transfer resistance of polymeric carbon nitride. It further indicates that Cl doping can promote transfer and separation of the photogenerated carriers [55], which agrees with the photoluminescence spectroscopy results and transient photocurrent response. The improved transport and separation can be affected by Cl atoms acting as a charge carrier transport bridge between the layers of carbon nitride [45,55].

The presented study revealed that the polycondensation of melamine with 2-chloro-4,6-diamino-1,3,5-triazine leads to formation of Cl-doped polymeric carbon nitride. The presented material showed improved photocatalytic properties in the hydrogen evolution reaction from water compared to pristine PCN. The investigation showed that the enhanced photocatalytic activity was attributed to an improved photogenerated charge transport and separation. Here, chlorine atoms could act as a charge carrier transport bridge between the carbon nitride layers. Although Cl doping did not affect the reduction in the bandgap energy, the transient photocurrent response of Cl-PCN was enhanced compared to pristine PCN, indicating a better transport and separation of the photoinduced charges. It indicates that a higher amount of electrons can migrate to the surface reaction sites before recombination leading to hydrogen evolution. These results are consistent with PL spectroscopy and EIS results, revealing a better separation and charge transfer, respectively, after chlorine doping. Moreover, it was revealed that chlorine doping affected the CB shift to a more negative potential, resulting in photogenerated electrons with a higher reducing ability in the water splitting reaction and, consequently, with a higher efficiency in hydrogen evolution.

## Conclusion

In summary, Cl-PCN nanosheets have been successfully synthesized via the polycondensation method. Fabricated 2D nanomaterials were used as photocatalysts for hydrogen evolution from water splitting. It was found that the Cl-modification had an effect on the photocatalytic efficiency. Also, main aspects were revealed: (i) a unique location of Cl atoms at the interlayers of PCN and not on its π-conjugated planes, (ii) a non-reduction in the bandgap energy, (iii) a lower recombination rate of the electron–hole pairs, (iv) improved photogenerated charge transport and separation, and (v) an enhanced reducing ability of the photogenerated electrons. Therefore, it is believed that heteroatom doping of pristine PCN is a suitable strategy towards boosting photocatalytic hydrogen evolution.

## Experimental

### Materials

All purchased reagents employed for catalyst preparation were analytical grade and used without further purification.

### Synthesis of polymeric carbon nitride

Polymeric carbon nitride was synthesized by direct heating of melamine. A given amount of melamine was placed in a covered crucible in a muffle furnace and heated in static air to 550 °C with a ramping rate of 4 °C/min and then was held at 550 °C for 4 h. After the reaction, the furnace was cooled down to room temperature. The obtained PCN was collected and milled into powder in an agate mortar.

### Synthesis of Cl-doped polymeric carbon nitride

As a precursor of chlorine, CDATA was used. Firstly, 4 g of melamine was mixed with 200 mg of CDATA in 20 mL of deionized water, upon stirring, for 1 h at room temperature. The solutions were then dried at 80 °C overnight. The obtained powders were ground in an agate mortar, placed into covered crucibles, and were submitted to thermal condensation under the same conditions as the PCN. The final products were milled in a mortar into a fine powder.

### Characterization

The morphology of the samples was analyzed using TEM (Tecnai F30) with an accelerating voltage of 200 kV. The FTIR spectra were recorded on a Nicolet 6700 FT-IR spectrometer. The chemical composition and relative atomic percentages on the surface of the samples were studied by XPS. The measurements were conducted using Mg Kα (*h*ν = 1253.6 eV) radiation in a Prevac (Poland) system equipped with a Scienta SES 2002 (Sweden) electron energy analyzer operating with a constant transmission energy (*E*_p_ = 50 eV). The analysis chamber was evacuated to a pressure below 5 × 10^−9^ mbar. The PL spectra were measured using a fluorescence spectrophotometer (F7000, Hitachi) with an excitation wavelength of 280 nm. The DRS was performed using a Jasco (Japan) spectrometer. The Kubelka–Munk function was used to calculate the bandgap energy. The photocurrent response and electrochemical impedance spectroscopy were measured using the Autolab PGSTAT302 N potentiostat in a three-electrode test cell with a platinum wire as the counter electrode and the saturated calomel electrode (SCE) as the reference. The working electrode was a fluorine-doped tin oxide (FTO) glass with the analyzed material drop-casted from a 0.2% ethanol/Nafion solution. A 0.5 M sodium sulfate solution was used as the electrolyte. The photocurrent (chronoamperometry) test was measured at 0.5 V vs SCE and the EIS test was conducted at 0.15 V vs SCE.

### Photocatalytic test

Prior to the photocatalytic test, each sample was prepared by dispersing 10 mg of the photocatalyst in 20 mL of water and sonicating for 1 h. The photocatalytic water splitting reaction was carried out in an outer irradiation-type reactor (Pyrex reaction vessel) connected to an argon source. After the reaction solution was placed in the reactor, 5 mL of lactic acid was poured into and purged with argon for air removal. Then, the reactor was irradiated with a Xe lamp (150 W) with an air mass filter (A.M. 1.5 G) to achieve a simulated solar light. The photocatalytic H_2_ evolution rate was analyzed by using a Young Lin 6500 gas chromatograph (GC, micro TCD detector, ValcoPLOT Molesieve 5 Å fused-silica column, and Ar as a carrier). Each catalyst was tested for 3 h. Every hour, 100 µL of gas was withdrawn from the reactor and injected into the gas chromatograph to measure the amount of H_2_ evolved.

## References

[1] Kudo A, Miseki Y (2009). Chem Soc Rev.

[2] Hisatomi T, Domen K (2019). Nat Catal.

[3] Chen X, Shen S, Guo L, Mao S S (2010). Chem Rev.

[4] Maeda K (2013). ACS Catal.

[5] Zhang H, Chen G, Bahnemann D W (2009). J Mater Chem.

[6] Di Paola A, García-López E, Marcì G, Palmisano L (2012). J Hazard Mater.

[7] Chatterjee D, Dasgupta S (2005). J Photochem Photobiol, C.

[8] Marszewski M, Cao S, Yu J, Jaroniec M (2015). Mater Horiz.

[9] Yu J, Wang K, Xiao W, Chenga B (2014). Phys Chem Chem Phys.

[10] Yu S, Jain P K (2019). ACS Energy Lett.

[11] Malato S, Fernández-Ibáñez P, Maldonado M I, Blanco J, Gernjak W (2009). Catal Today.

[12] Robertson P K J, Robertson J M C, Bahnemann D W (2012). J Hazard Mater.

[13] Shiraishi Y, Hirai T (2008). J Photochem Photobiol, C.

[14] Lang X, Chen X, Zhao J (2014). Chem Soc Rev.

[15] Niu P, Zhang L, Liu G, Cheng H-M (2012). Adv Funct Mater.

[16] Zheng Y, Lin L, Wang B, Wang X (2015). Angew Chem, Int Ed.

[17] Reddy K R, Reddy C V, Nadagouda M N, Shetti N P, Jaesool S, Aminabhavi T M (2019). J Environ Manage.

[18] Ong W-J, Tan L-L, Ng Y H, Yong S-T, Chai S-P (2016). Chem Rev.

[19] Zhang J, Sun J, Maeda K, Domen K, Liu P, Antonietti M, Fu X, Wang X (2011). Energy Environ Sci.

[20] Tan G, She L, Liu T, Xu C, Ren H, Xia A (2017). Appl Catal, B.

[21] Wang Y, Wang X, Antonietti M (2012). Angew Chem, Int Ed.

[22] Yu W, Xu D, Peng T (2015). J Mater Chem A.

[23] Zhou S, Liu Y, Li J, Wang Y, Jiang G, Zhao Z, Wang D, Duan A, Liu J, Wei Y (2014). Appl Catal, B.

[24] Wang L, Hong Y, Liu E, Wang Z, Chen J, Yang S, Wang J, Lin X, Shi J (2020). Int J Hydrogen Energy.

[25] Kang Y, Yang Y, Yin L-C, Kang X, Liu G, Cheng H-M (2015). Adv Mater (Weinheim, Ger).

[26] Yan S C, Li Z S, Zou Z G (2010). Langmuir.

[27] Lan Z-A, Zhang G, Wang X (2016). Appl Catal, B.

[28] Liao G, Gong Y, Zhang L, Gao H, Yang G-J, Fang B (2019). Energy Environ Sci.

[29] Reddy N L, Kumbhar V S, Lee K, Shankar M V, Pandikumar A, Rameshkumar P (2020). Graphitic carbon nitride–based nanocomposite materials for photocatalytic hydrogen generation. Nanostructured, Functional, and Flexible Materials for Energy Conversion and Storage Systems.

[30] Wang Y, Di Y, Antonietti M, Li H, Chen X, Wang X (2010). Chem Mater.

[31] Hong J, Hwang D K, Selvaraj R, Kim Y (2019). J Ind Eng Chem (Amsterdam, Neth).

[32] Jing L, Zhu R, Phillips D L, Yu J C (2017). Adv Funct Mater.

[33] Dang X, Yang R, Wang Z, Wu S, Zhao H (2020). J Mater Chem A.

[34] Ma X, Lv Y, Xu J, Liu Y, Zhang R, Zhu Y (2012). J Phys Chem C.

[35] Wang K, Li Q, Liu B, Cheng B, Ho W, Yu J (2015). Appl Catal, B.

[36] Ding K, Wen L, Huang M, Zhang Y, Lu Y, Chen Z (2016). Phys Chem Chem Phys.

[37] Hu C, Hung W-Z, Wang M-S, Lu P-J (2018). Carbon.

[38] Wang H, Wang B, Bian Y, Dai L (2017). ACS Appl Mater Interfaces.

[39] Yi F, Gan H, Jin H, Zhao W, Zhang K, Jin H, Zhang H, Qian Y, Ma J (2020). Sep Purif Technol.

[40] Liu J, Huang J, Zhou H, Antonietti M (2014). ACS Appl Mater Interfaces.

[41] Chen S, Duan J, Tang Y, Zhang Qiao S (2013). Chem – Eur J.

[42] Zhao D, Chen J, Dong C-L, Zhou W, Huang Y-C, Mao S S, Guo L, Shen S (2017). J Catal.

[43] Wang N, Fan H, Sun J, Han Z, Dong J, Ai S (2016). Carbon.

[44] Han E-X, Li Y-Y, Wang Q-H, Huang W-Q, Luo L, Hu W, Huang G-F (2019). J Mater Sci Technol.

[45] Liu C, Zhang Y, Dong F, Reshak A H, Ye L, Pinna N, Zeng C, Zhang T, Huang H (2017). Appl Catal, B.

[46] Baca M, Rychtowski P, Wróbel R, Mijowska E, Kaleńczuk R J, Zielińska B (2020). Sol Energy.

[47] Guo F, Shi W, Zhu C, Li H, Kang Z (2018). Appl Catal, B.

[48] Zheng Y, Liu J, Liang J, Jaroniec M, Qiao S Z (2012). Energy Environ Sci.

[49] Wang X, Blechert S, Antonietti M (2012). ACS Catal.

[50] Liu J, Liu Y, Liu N, Han Y, Zhang X, Huang H, Lifshitz Y, Lee S-T, Zhong J, Kang Z (2015). Science.

[51] Kuriki R, Sekizawa K, Ishitani O, Maeda K (2015). Angew Chem, Int Ed.

[52] Wang X, Maeda K, Thomas A, Takanabe K, Xin G, Carlsson J M, Domen K, Antonietti M (2009). Nat Mater.

[53] Guo F, Shi W, Wang H, Huang H, Liu Y, Kang Z (2017). Inorg Chem Front.

[54] Su F, Mathew S C, Möhlmann L, Antonietti M, Wang X, Blechert S (2011). Angew Chem, Int Ed.

[55] Long D, Diao W, Rao X, Zhang Y (2020). ACS Appl Energy Mater.

[56] Hong J, Xia X, Wang Y, Xu R (2012). J Mater Chem.

[57] Feng J, Zhang D, Zhou H, Pi M, Wang X, Chen S (2018). ACS Sustainable Chem Eng.

[58] Mishra B P, Babu P, Parida K (2021). Mater Today: Proc.

[59] Guo S, Tang Y, Xie Y, Tian C, Feng Q, Zhou W, Jiang B (2017). Appl Catal, B.

[60] Long D, Chen W, Zheng S, Rao X, Zhang Y (2020). Ind Eng Chem Res.

[61] Liu G, Niu P, Sun C, Smith S C, Chen Z, Lu G Q, Cheng H-M (2010). J Am Chem Soc.

[62] Thaweesak S, Wang S, Lyu M, Xiao M, Peerakiatkhajohn P, Wang L (2017). Dalton Trans.

[63] Chen P, Xing P, Chen Z, Lin H, He Y (2018). Int J Hydrogen Energy.

[64] Zeng Y, Liu X, Liu C, Wang L, Xia Y, Zhang S, Luo S, Pei Y (2018). Appl Catal, B.

[65] Zhu B, Zhang J, Jiang C, Cheng B, Yu J (2017). Appl Catal, B.

[66] Dong G, Zhao K, Zhang L (2012). Chem Commun.

[67] Han Q, Hu C, Zhao F, Zhang Z, Chen N, Qu L (2015). J Mater Chem A.

[68] Zhang G, Zhang M, Ye X, Qiu X, Lin S, Wang X (2014). Adv Mater (Weinheim, Ger).

[69] Guo Y, Chen T, Liu Q, Zhang Z, Fang X (2016). J Phys Chem C.

[70] Zhao S, Zhang Y, Wang Y, Zhou Y, Qiu K, Zhang C, Fang J, Sheng X (2017). J Power Sources.

[71] Bu Y, Chen Z, Li W (2014). Appl Catal, B.

